# Endodontic Management of Mandibular Second Premolar with Vertucci Root Canal Configuration Type V

**DOI:** 10.1155/2022/3197393

**Published:** 2022-04-01

**Authors:** Shahzad Ali Shah

**Affiliations:** Department of Restorative Dentistry, College of Dentistry in Ar-Rass, Qassim University, Saudi Arabia

## Abstract

The diversity of root canal anatomy in permanent dentition is quite common. Understanding the basic anatomy of the root canal morphology and its variations is particularly important for successful root canal treatment. Mandibular second premolar usually consists of a single root with a single root canal. The presence of split roots in the apical third of the root with two separate root canals is quite rare. The major cause of endodontic failure in missed canals is that it harbors bacteria and other microorganisms. Careful radiographic interpretation and clinical examination of the pulp chamber will be helpful in locating canal orifices. The mandibular premolars with Vertucci type V canal configuration pose a challenge in clinical management. Identifying them early is important to aid appropriate modification in treatment protocol. In this case report, important modifications of clinical steps and application of magnification for successful management with access chamber modification are explained.

## 1. Introduction

Success in root canal treatment lies in a thorough knowledge of root canal systems and their three-dimensional cleaning and shaping followed by hermetic sealing of the cleaned and shaped canals ([1], 83-95). Premolars are unique in the way that there have been a lot of anatomical variations in the root canal system and number of roots. Lack of knowledge and understanding regarding anatomical variation may result in untreated canals, potentially leading to root canal treatment failure ([2], 430-435). Pulp space is complex, the canals may divide, rejoin, and divide again and possess forms that may not be commonly employed. Many teeth have additional roots or additional canals and a variety of canal configurations.

The mandibular premolars typically present with single root and single canal which constitute Type I of Vertucci canals classification ([3], 47-50). The single root is usually oval shaped with an oval cross-section canal all the way to the apex. Several studies have shown that mandibular second premolar has nonsingle canal at the apex in 2—9.9% of the cases ([4], 1448-1452; [5], 1007-1012; [6], 931-941; [7], 1096-1104). These significant canal configuration variations are caused by differences in sex, age, ethnicity, and race ([8], e0165329) (Table 1).

Diagnosis of root canal variation is important and different tools are employed over the years to tackle this challenge with various shortcomings. Clinically, enhanced visual aids such as digital operating microscope are one of the noninvasive methods which offers magnified view and clear visualization of the site. Recently, the addition of CBCT (Cone Beam Computed Tomography) as a diagnostic tool has increased the success of accurate diagnosis to greater levels ([9], 8170620-8).

Literature review of this subject reveals greater variations and diversities in the root canal system of the mandibular premolars ([10], 2615746-6; [11], 4495). Burkan and Bhuwan ([12], bcr-222563; [13] a, 72-75) reported premolars with taurodontism and multiple canals. Other studies have reported variations in number of roots, canals, and presence of root bifurcation. The single-rooted mandibular premolars have anatomy that usually is not continuous from the canal orifice to the apical foramen. The clinically important characteristic is the presence of isthmuses that connect the individual canals and run across the bifurcations. Cleaning and shaping of these isthmuses are a clinical challenge that requires a carefully devised treatment plan and clinical knowledge to avoid procedural errors. Vertucci reported that second premolars have only one root canal at the apex in 97.5% of the teeth understudy and two canals were only found in 2.5% of cases; the incidence of three root canals was extremely rare ([3], 47-50). Aricioglu et al. ([14], 443-451) have reported a case of taurodont second premolar and some others documented cases of taurodont molars ([15], 344-347)..

This case reports a successful root canal treatment of mandibular second premolar with a single root that divides into two in the apical third area making it Vertucci configuration Type V.

## 2. Case Report

A 20-year-old female patient visited Out Patient Department of the Dental Clinics of College of Dentistry in Ar-Rass, Qassim University, with the chief complaint of pain on percussion in the right mandibular premolars, for about 2 months. Medical history was noncontributory.

Clinical examination revealed moderate oral hygiene. There was bleeding on probing but no periodontal pockets. The tooth involved was painful to percussion. From the clinical examination, it was revealed that the patient has a high-risk carious profile with more than three active carious lesions at the time of visit. There was a distal class II cavity in tooth #15 and tooth #26. Tooth #36 and 44 had a class II cavity and tooth #35 and #45 were previously restored. Both teeth (35 and 45) were painful to percussion.

**Figure 1:**
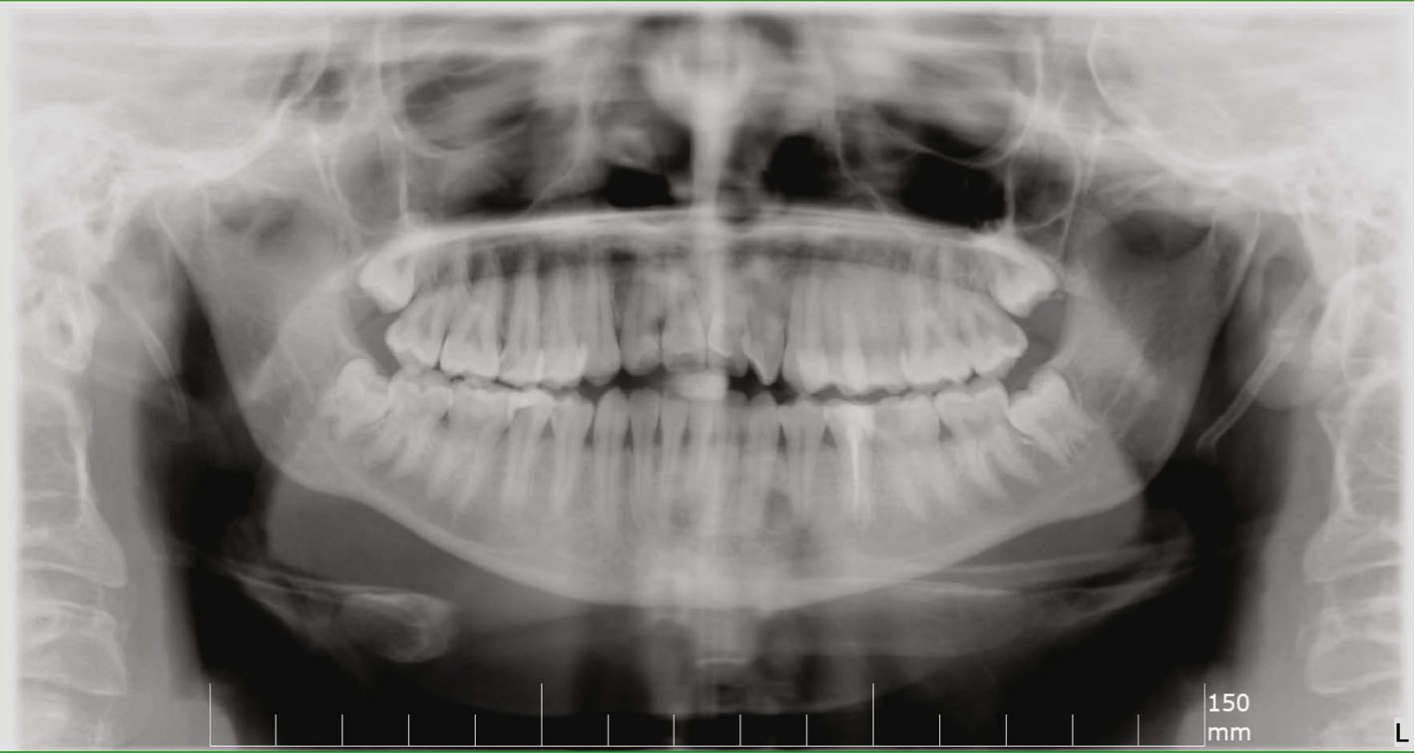
Survey radiograph showing tooth #35 endo treated and #45 with periapical radiolucency. The apical third of both tooth roots has aberrated canals.

On OPG radiographic examination, all third molars were unerupted. Tooth #45 had periodontal widening with periapical radiolucency later confirmed with periapical radiograph. Tooth #35 was previously root canal treated with inadequate obturation. All other findings on radiographs matched the clinical examination (Figure 1).

**Figure 2:**
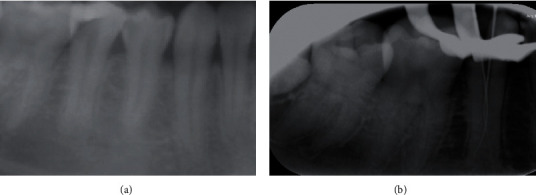
(a) Radiograph showing tooth #45 with sudden loss of canal continuation and root bifurcation and (b) working length periapical radiograph clearly showing two separate canals in the apical portion.

**Figure 3:**
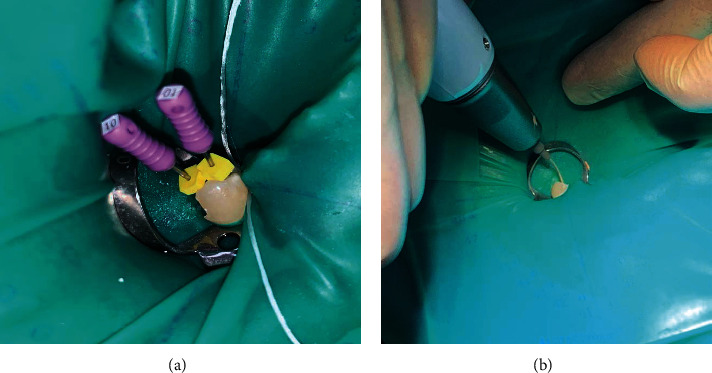
(a) Root canal length determination using #10 SS files (K-FILES, MANI, Tochigi, Japan). (b) Root canal filling using thermoplastic technique with DIA-DUO (DiaDent Group International Inc).

Based on the clinical and survey radiographic examinations, the patient with informed consent was referred to the radiology department for further investigation. Tooth #45 was focused in periapical radiograph using the DIGORA digital system (SOREDEX™ DIGORA™ Optime, intraoral digital X-ray). Periapical radiograph revealed sudden loss of canal continuity and increase in the canal mesiodistal width at the apical third area. A suspicion of canal aberration was noted (Figure 2(a)), subsequent to confirmatory diagnosis of pulpal necrosis with symptomatic apical periodontitis. The treatment plan has been fully explained to the patient, which includes complete caries removal, access cavity preparation, cleaning, and shaping followed by intracanal medication in the first visit. The second visit will include root canal filling if asymptomatic followed by postplacement and composite buildup. Complications during treatment and chances of root canal treatment success and failure were discussed. Following patient's informed consent, local mandibular block anesthesia (1.7 ml lidocaine HCL 2% and Epinephrine 1 : 100,000 USP injection) was incorporated, a dental dam was placed on tooth #45 and the carious lesion was removed completely followed by access cavity preparation. Careful visual inspection of the pulp chamber was performed using the SLF dental magnification loupes (6X-420, Zumax Medical Co. Ltd) with additional attached (LED) light that revealed oval-shaped single canal orifice at the Cemento Enamel Junction (CEJ) level. The pulp chamber was cleaned of necrotic tissue using 3 ml of 2.5% concentration of sodium hypochlorite followed by using an endodontic explorer (DG-16) that was inserted to inspect the canal bifurcation at the apical level. The access cavity was modified using Premier Glades Glidden drills of size 1-5 (Premier Dental Co.) for coronal flaring and to get better access to the separated canals at the apical portion and establish a glide path. A mesiolingually precurve SS #10 K-file (K-FILES, MANI, Tochigi, Japan) was placed in the canal orifice. Another file with #10 K-files (K-FILES, MANI, Tochigi, Japan) was placed in the canal with distobuccal direction and the canal length was preliminarily taken using an apex locator (Root ZX mini, Morita, Japan) which later confirmed with periapical radiographs (Figures 2(b) and 3(a)).

After confirmation of the root length and initial canal preparation till file # ISO 25 K-files (K-FILES, MANI, Tochigi, Japan), the canals were cleaned and shaped using the Pro-Taper Universal (Dentsply Maillefer) up to file #F2 (25/.04) with copious irrigation using 5 ml of warm 2.5% sodium hypochlorite using side-vented endodontic irrigation needles. 2 ml of 17% ethylenediaminetetraacetic acid (EDTA) was used as a last rinse to remove smear layer followed by rinsing with normal saline. Canals were dried using paper points and filled with calcium hydroxide (TYPE I, GUANYA, Wuhan, China) paste using the Lentulo spiral (Dentsply Maillefer) size 25. The access chamber was sealed with Cavit (3 M ESPE) for one week. Patient was discharged and Panadol Extra tablets (Paracetamol+ caffeine 500 mg/65 mg) were prescribed twice daily for 5 days.

**Figure 4:**
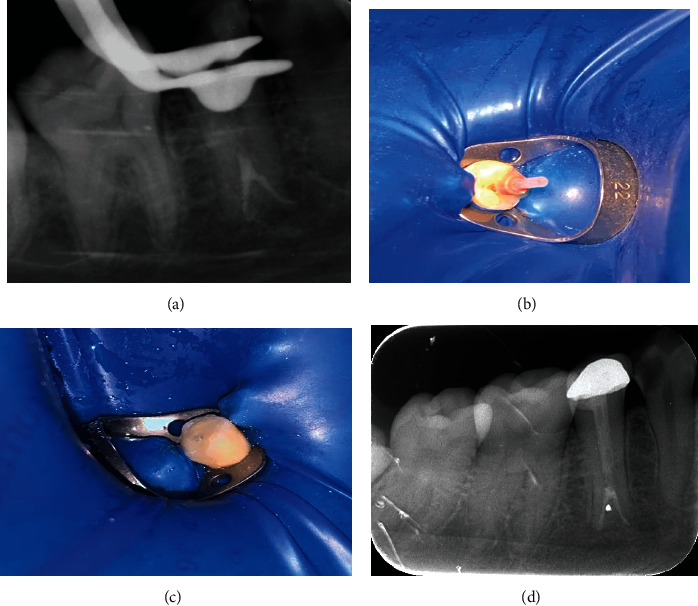
Final filling of tooth #45. (a) Periapical radiograph showing final filling of the root canals. Space was created for fiber postcementation. (b) Photographic view of using fiber postinsertion and cementation. (c) Crown placement. (d) A follow-up radiograph after one year showing resolution of periapical lesion. (Note progression of caries in tooth #44).

On the second appointment, clinical examination showed the tooth was completely asymptomatic. After application of local anesthesia (1.7 ml lidocaine HCL 2% and Epinephrine 1 : 100,000 USP injection) and dental dam isolation, temporary filling was removed and the access cavity was carefully inspected. Calcium hydroxide was removed using SS file #25 and sodium hypochlorite irrigation solution. Canals were dried using paper points and filling was performed with thermoplastic filling technique using DIA-DUO (DiaDent Group International Inc) and AH plus (Dentsply Maillefer) sealer, followed by thermoplastic gutta-percha backfill (Figure 3(b)). Periapical radiograph was taken after completion of the root canal filling (Figure 4(a)).

**Figure 5:**
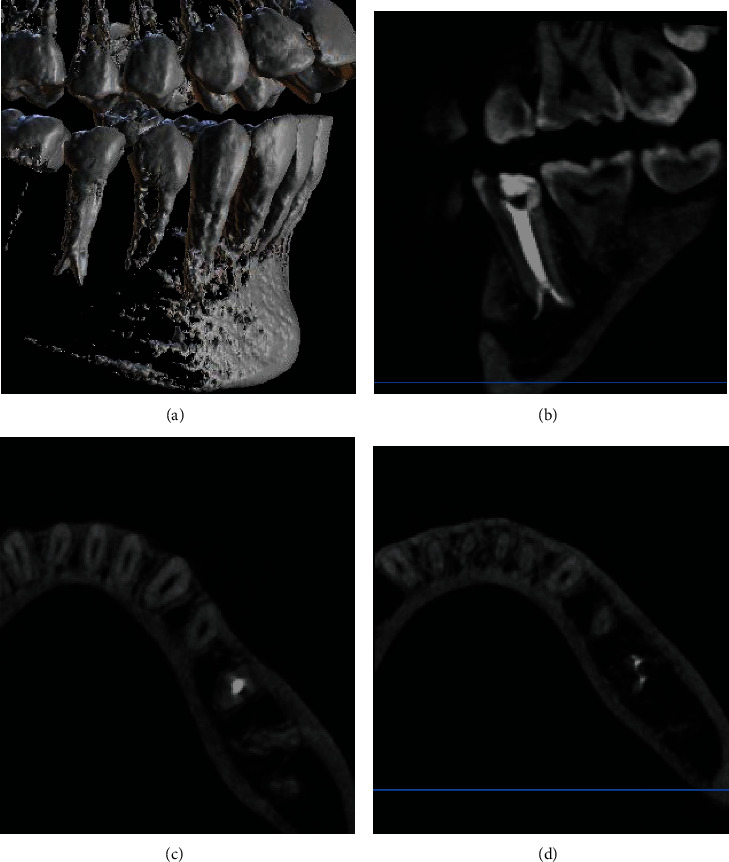
CBCT scans. (a) 3-D reconstruction (Blue Sky Plan 4 software) of tooth #45 showing root bifurcation of apical third area. (b) Sagittal section showing root filling of the premolar with canal bifurcation in apical third. (Note: periapical radiolucency connecting with mental foramen). (c) Occlusal view showing filling of the midportion of the canal. (d) Occlusal view of the apical third of the root showing two separate canals duly filled.

After 2 weeks of observation, the patient showed no spontaneous pain and no obvious abnormalities in the buccal or lingual mucosa. It was decided to take a preoperative CBCT scan ([16], 1675-1678) for previously treated tooth #35 with poor canal fillings to assess the canal variation in detail, due to lack of information about the variation in canal morphology. Advance imaging tools such as CBCT proved to be an additional diagnostic and management tool in revealing external and internal canal morphology. This CBCT (SOREDEX-CRANEX® 3D) was additionally used in transverse, sagittal, and axial planes to assess the final filling of tooth #45 with 3-dimensional seal with field of view 6 × 8 cm (high resolution) and a voxel size of 0.20 mm. The Cone Beam Computed Tomography scan (SOREDEX-CRANEX® 3D) was visualized using the OnDemand 3DApp-3D software. (Figure 5).

Coronal two third of the filling material was removed and the coronal portion was prepared for a fiber post cementation using the Rely X (RelyX™ Unicem Self-Adhesive Universal Resin Cement Aplicap™/Maxicap™, 3 M ESPE) and coronal composite buildup (Filtek Z350 XT, 3 M ESPE) (Figure 4(b)). The following week, the patient was recalled for crown preparation and cementation (Figure 4(c)). A follow-up radiograph was taken after one year (Figure 4(d)).

## 3. Discussion

Diagnosis of root canal variability has been the subject of research since recent advancements in diagnostic equipment in dentistry ([17], 93; Chaintiou Piorno et al. 2021, 105040; [18], 263-269). Success of the root canal treatment highly demands meticulous clinical examination and judicial use of diagnostic aids followed by thorough cleaning/shaping and filling of all the canals. Inability to locate, debride, and fill all canals has been reported to be the major cause of failure for root canal treatment ([19], 834-836). The mandibular premolars have been reported to have the reputation of variable anatomy ([20], 1216-1221). Multiple studies have reported the mandibular premolars to have more than one canal ([21], 538-541; [22], 1410-1416). Arayasantiparb et al. ([23], 201-207) reported in their study an incidence of multiple root canals in the mandibular first premolar as 19.48% and in second premolar 3.85%. The majority of multiple root canals were defined as Vertucci's type V (1–2 canals). If these studies are kept in mind, endodontists are always recommended to look for the missed canal.

Conventional radiographs depict a two-dimensional picture of a three-dimensional object, which usually results in superimposition of images. Therefore, their use and value are rather limited in complex root canal morphology cases. Interpretation based on conventional radiographs may alarm the clinician to the presence of unusual anatomy but usually cannot fully show the exact picture of canal morphology and their interrelations. (Anonymous 2020, 399).

Rapid (fast) break is the term used when there is a sudden discontinuity of the canal on the radiograph. This usually happens when the main canal divides into two or more branches. The canal discontinuity in this case was located in the apical third of the root. It was difficult to locate the canal clinically due to poor vision and low brightness. Therefore, the middle third of the canal was expanded using the Gates Glidden drills, and the root canals were examined by a SLF dental surgical loupe with 6 × 420 magnification. An additional overhead light source was attached to the loupe frame to enhance the vision and helped locate the canals thus enhancing magnification and reducing chances of procedural errors.

Currently, the literature commonly reports the number of root canals of the first mandibular premolar to be 1–4, and some cases have type C roots. According to the standard classification by Vertucci, ([24], 589-599) statistical results show a consistent global trend in the morphology of root canal of the mandibular first premolars whereas, most have one canal, some have two root canals, and cases of three or more canals are rare.

## 4. Conclusion

This case report contributes to the understanding of the variation in the root canal anatomical structure of the mandibular premolar teeth, resulting in clinicians finding another reference to similar case studies.

### 4.1. Suggestions for Improvement

More advanced clinical protocols of root canal treatment should be used with recently available biofriendly sealers.

A Small FOV (5 mm or less) for CBCT will yield better results of diagnostic accuracy.

Long-term follow-up of up to 4 years will be ideal in this case.

## Figures and Tables

**Table 1:** Review table of the prevalence of variations in root anatomy and canal configuration of human permanent mandibular premolars.

Number of roots	Root canal anatomy	Diagnostic mode	Country	References
1 root	3 canals 5 canals	R/G R/G	India Argentina	([25], 392-394) ([26], 304-305)
2 roots	2 canals 5 canals 3 canals	R/G R/G CBCT	India Turkey Iran	([27], 70-73) ([28], 81-84) ([29], 25-28)
3 roots	3 canals 4 canals	Spiral CT R/G	India India	([30], 816576-4) ([13], 72-75)

## Data Availability

The (clinical pictures and radiographs) data used to support the findings of this study are included within the article. A separate editable PowerPoint file is added for edit.
